# Supplementary material for: Development and Optimisation of HILIC-LC-MS Method for Determination of Carbohydrates in Fermentation Samples
Source: Molecules. 2021 Jun 16;26(12):3669. doi: 10.3390/molecules26123669 (PMC8235229; doi:10.3390/molecules26123669)
Supplement: Supplementary file 1 [file molecules-26-03669-s001.zip › molecules-1211337-supplementary.pdf]

Supplementary Materials

Development and Optimisation of HILIC-LC-MS Method for Determination of Carbohydrates in Fermentation Samples

Dmitri Pismennõi [1,2,*], Vassili Kiritsenko [1,2], Jaroslav Marhivka [1,2], Mary-Liis Kütt [1] and Raivo Vilu [1]

[1] Center of Food and Fermentation Technologies (TFTAK), Akadeemia tee 15A, 12618 Tallinn, Estonia
[2] Department of Chemistry and Biotechnology, Tallinn University of Technology, Akadeemia tee 15, 12618 Tallinn, Estonia

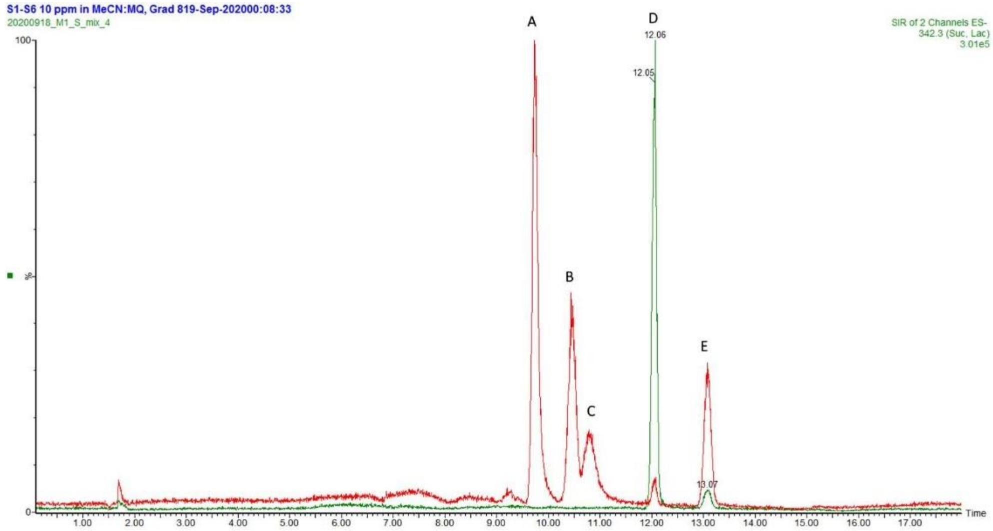

Figure S1. The chromatogram of 5 saccharides subjected to the optimised gradient elution program with flow rate of 300 µL/min. The peaks are labelled as follows: A – fructose, B – glucose, C-galactose,D– sucrose, E – lactose. Peak heights are normalised.

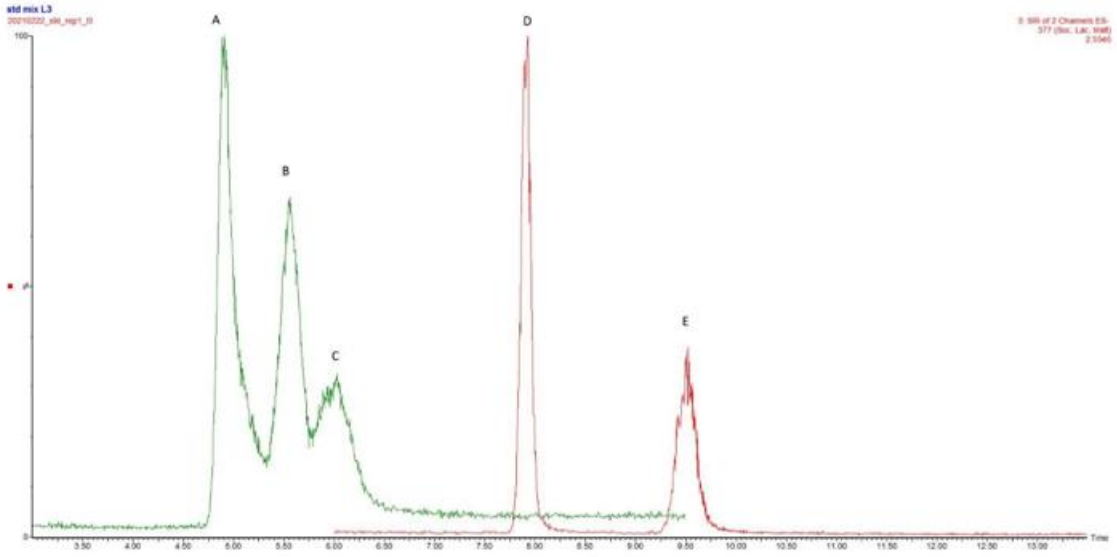

Figure S2. The chromatogram of 5 saccharides subjected to the optimised gradient elution program with flow rate of 313 µL/min. The peaks are labelled as follows: A – fructose, B – glucose, C- galactose,D– sucrose, E – lactose. Peak heights are normalised.

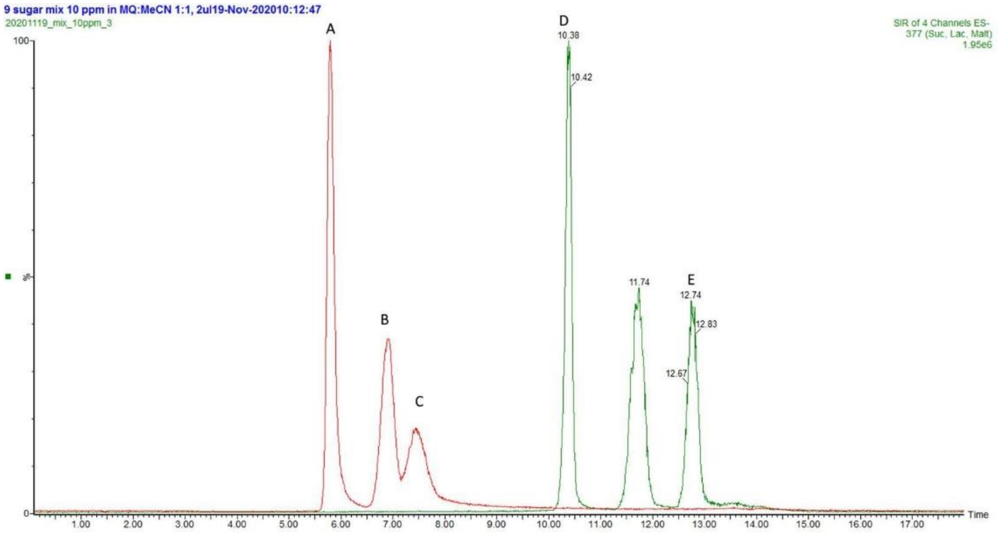

Figure S3. The chromatogram of 5 saccharides subjected to the optimised gradient elution program with flow rate of 350 µL/min. The peaks are labelled as follows: A – fructose, B – glucose, C-galactose,D– sucrose, E – lactose. Peak heights are normalised.

Table S1. The peak areas of analytes while using neat solvents or solvents with added guanidine hydrochloride. The gradient programm and mass spectrometric parameters, except for SIR channels,remained the same across all runs.

	**Neat Solvents**	**Addition of Guanidine Hydrochloride**	**Difference, %**
Fructose	3.08×10^5	6.35×10^5	206
Glucose	1.64×10^5	3.25×10^5	198
Galactose	1.18×10^5	3.59×10^5	304
Sucrose	2.04×10^5	3.91×10^5	191
Lactose	7.92×10^4	2.17×10^5	274

Table S2. The peak areas of five saccharides in this study subjected to different extraction protocol in order to perform the sample preparation optimisation. The chromatographic and mass spectrometricparameters remaind the same across (Table 1 in the main text of the article).

	**Protocol nr.1**	**Protocol nr.2**	**Protocol nr.3**	**Protocol nr.4**	**Protocol nr.5**	**Protocol nr.6**
Fructose	7.594×10^4	2.039×10^4	4.004×10^3	2.136×10^3	8.041×10^4	7.411×10^4
Glucose	2.785×10^5	1.832×10^5	1.587×10^5	1.419×10^5	2.809×10^5	2.446×10^5
Galactose	6.040×10^4	1.474×10^4	Not determined	Not determined	5.999×10^4	5.934×10^4
Sucrose	1.399×10^5	3.084×10^4	1.057×10^4	4.813×10^3	1.332×10^5	1.195×10^5
Lactose	2.509×10^5	2.176×10^5	2.013×10^5	1.868×10^5	2.441×10^5	2.104×10^5
